# The Predictive Role of Red Cell Distribution Width in Mortality among Chronic Kidney Disease Patients

**DOI:** 10.1371/journal.pone.0162025

**Published:** 2016-12-01

**Authors:** Yao-Peng Hsieh, Chia-Chu Chang, Chew-Teng Kor, Yu Yang, Yao-Ko Wen, Ping-Fang Chiu

**Affiliations:** 1 Division of Nephrology, Department of Internal Medicine, Changhua Christian Hospital, Changhua, Taiwan; 2 Ph.D. program in translational medicine, College of Life Science, National Chung Hsing University, Taichung, Taiwan; 3 Kaohsiung Medical University, Kaohsiung, Taiwan; 4 School of Medicine, Chung Shan Medical University, Taichung, Taiwan; Chang Jung Christian University, TAIWAN

## Abstract

**Background:**

Recently, accumulating evidence has demonstrated that RDW independently predicts clinically important outcomes in many populations. However, the role of RDW has not been elucidated in chronic kidney disease (CKD) patients. We conducted the present study with the aim to evaluate the predictive value of RDW in CKD patients.

**Methods:**

A retrospective observational cohort study of 1075 stage 3–5 CKD patients was conducted in a medical center. The patients’ baseline information included demographic data, laboratory values, medications, and comorbid conditions. The upper limit of normal RDW value (14.9%) was used to divide the whole population. Multivariate Cox regression analysis was used to determine the independent predictors of mortality.

**Results:**

Of the 1075 participants, 158 patients (14.7%) died over a mean follow-up of approximately 2.35 years. The crude mortality rate was significantly higher in the high RDW group (high RDW group, 22.4%; low RDW group 11%, p <0.001). From the adjusted model, the high RDW group was correlated with a hazard ratio of 2.19 for overall mortality as compared with the low RDW group (95% CI = 1.53–3.09, p<0.001). In addition, the high RDW group was also associated with an increased risk for cardiovascular disease (HR = 2.28, 95% CI = 1.14–4.25, p = 0.019) and infection (HR = 1.9, 95% CI = 1.15–3.14, p = 0.012)) related mortality in comparison with the low RDW group.

**Conclusions:**

In stage 3–5 CKD patients, RDW was associated with patient mortality of all-cause, cardiovascular disease and infection. RDW should be considered as a clinical predictor for mortality when providing healthcare to CKD patients.

## Introduction

Anemia is a common complication for chronic kidney disease (CKD) patient and is correlated with increased risk of mortality and hospitalization. Decreased erythropoietin secretion from the dysfunctional kidney is the major feature in renal anemia.

Red cell distribution width (RDW) is the measurement of the variation in circulatory erythrocyte size and is routinely reported as a part of complete blood cell counts at no additional cost. It has been commonly used, in conjunction with mean corpuscular volume, as one index to narrow the differential diagnosis of anemia. Higher RDW refers to a greater heterogeneity in RBC size (anisocytosis). Elevated RDW may point to alteration in the erythrocyte life span due to impaired production or increased destruction of erythrocytes. Apart from its role in anemia, RDW has been recently found to be a novel and independent predictor for mortality in the general population, as well as in patients with chronic heart failure, peripheral artery disease, and kidney transplants [1–4]. RDW has also been reported to be correlated with patient survival in acute clinical settings, including acute myocardial infarction, acute pulmonary embolism, acute heart failure, pneumonia and acute kidney injury treated with continuous renal replacement therapy [5–9]. It is also a useful marker in the risk stratification of contrast induced acute kidney injury [10].

CKD is considered a status of increased inflammation and oxidative stress and endothelial dysfunction. A disproportionately high cardiovascular disease burden has been attributed to these untraditional risk factors. Since RDW has been found to be associated with endothelial dysfunction that leads to adverse impact in patients with chronic kidney disease [11], we aimed to test the hypothesis that RDW is directly correlated with clinical outcomes, including all-cause, cardiovascular disease, and infection related mortality in stage 3 to 5 CKD patients.

## Patients and Methods

We carried out a retrospective cohort investigation at a Taiwanese medical center using the established computerized data and electronic medical records from 2006 to 2012. Eligible participants for the study were those who joined the integrated CKD care program between 2006 and 2011. The definition of CKD diagnosis was based on the National Kidney Foundation Kidney Disease Outcomes Quality Initiative (KDOQI) criteria. We estimated the baseline glomerular filtration rate (eGFR) with the following the Modification of Diet in Renal Disease (MDRD) study equation: eGFR ml/min per 1.73 m^2^ = 186×serum creatinine^-1.154^ ×age^-0.203^×0.742 (if female patient) ×1.212 (if black patient). The exclusion criteria included: stage 1–2 CKD (n = 257), or subjects aged under 20 years (n = 8) or more than 80 years (n = 6). or those were lost to follow-up in 3 months (n = 72).Finally, our study cohort comprised 1075 stage 3–5 CKD patients. All the study participants were followed till death, or the study end on December 31. The study protocol was approved by the institutional review board of Changhua Christian Hospital (CCH-IRB- 150903) and conducted in compliance with the declaration of Helsinki. The written informed consent for each participant was not required for such a retrospective cohort study in Taiwan. All the patient records or information was anonymized and de-identified prior to analysis.

The baseline patient characteristics included age, sex, body mass index, educational level, the etiology of CKD, comorbidities, medications and blood tests. The comorbid conditions comprised diabetes mellitus (DM), hypertension, coronary artery disease, congestive heart failure, cerebrovascular and peripheral artery disease, cancer, dementia and chronic lung disease, liver cirrhosis, hyperlipidemia. The laboratory parameters included blood levels of blood urea nitrogen (BUN), creatinine, albumin, white blood cell (WBC) counts, platelet counts, mean corpuscular volume (MCV), hemoglobin, red cell distribution width (RDW), cholesterol, triglyceride, uric acid, calcium, phosphate, and 24-hour proteinuria. The pharmacotherapy history encompassed angiotensin-converting enzyme (ACE) inhibitors, angiotensin II receptor blockers (ARB), anti-anemia agents (iron preparation, folic acid, and vitamin B12), erythropoiesis stimulating agents, calcium channel blocker (CCB), and phosphate binders. Measurement of red blood cell parameters was carried out using the automatic hematology analyzer (DxH 800, Beckman Coulter). The normal reference range was 11.7%~ 14.9% for RDW at our laboratory. For the analysis of the predictive effect of RDW on outcomes of interest, subjects were divided into two groups by the upper normal limit of the RDW value as: the high RDW group, >14.9%; the low RDW group: ≦14.9%. The two most common causes of mortality in our cohort were CVD and infection. The primary outcomes of this study were all-cause mortality, infection associated mortality and CVD mortality.

## Statistical analysis

All the data are presented as mean ± standard deviation (SD) for continuous variables, or number (n) and percentage (%) for categorical variables, as appropriate. The differences among the groups were assessed using the Chi-square test or Fisher’s exact test for categorical variables. Comparisons of continuous variables were done using a student t test or Mann-Whitney test, as appropriate. Kaplan-Meier survival curves were generated to compare the survival differences between the high RDW group and the low RDW group and the log-rank test was performed to assess the significance. The Cox proportional hazards model was used to determine whether RDW was a significant predictor for mortality. The covariates for adjustments included gender, age, BMI, comorbid conditions, blood levels of cholesterol, triglyceride, BUN, creatinine, estimated GFR, albumin, calcium, phosphate, the Ca x P product, hemoglobin, proteinuria, WBC counts, platelet counts, MCV, uric acid, and the medications (anti-anemia agents, erythropoiesis stimulating agents, CCB, phosphate binders, ACE inhibitor, and angiotensin II receptor blocker).

To assess the predictive power of RDW for the clinical outcomes, four models for adjustments of covariates were done: model 1, adjusted for sex, age, BMI and smoking status; model 2, adjusted for all variables in model 1, plus laboratory parameters; model 3, adjusted for all variables in model 2, plus pharmacologic treatment; model 4, adjusted for all variables in model 3, plus comorbid conditions. Spearman rank correlation test was used to determine the relation between RDW and other parameters. All the statistics were two-sided tests and the results were considered significant for a p value of less than 0.05. All statistical analyses were done using the statistical package for Windows, SAS 9.2 (SAS Institute Inc, Cary, NC, USA) and SPSS 16.0 (SPSS, Chicago, IL, USA).

## Results

### Baseline characteristics of the study cohort

Baseline demographic and clinical data of the 1075 study subjects are shown in Table 1. The mean age of the cohort was 64.2 ±12.35 years and 56.28% were men. The mean follow-up duration was 28.2 ± 19.8 months. Among the participants, diabetes mellitus (39.4%) and hypertension (23.8%) accounted for the two most common causes of CKD. Most of the participants were not current smokers and received on primary school educational levels. Additional information stratified by the RDW categories (the low and high RDW group) is listed in Table 2. Patients in the high RDW group were older (65.77 ± 11.45 vs. 63.44 ± 12.71 years, p = 0.003). Overall, the prevalence of comorbid conditions was comparable between the high RDW and low RDW group, except a higher prevalence of congestive heart failure and coronary artery disease in the high RDW group. Furthermore, the high RDW group also had higher levels of BUN, creatinine, WBC counts, and phosphorus, and lower levels of albumin, eGFR, calcium, hemoglobin, and MCV while compared with the low RDW group. Concerning the medications, the high RDW group had a higher proportion of prescribed iron preparations, folic acid, erythropoiesis stimulating agents and phosphate binders.

**Table 1:** Baseline characteristics of study population

Variable	All Subjects
	(n = 1075)
sex (% male)	605(56.28%)
Age (years)	64.2±12.35
BMI (kg/ m^2^)	25.17±4.15
follow-up duration (months)	28.21±19.84
CKD at study enrollment	
Stage 3	304(28.28%)
Stage 4	403(37.49%)
Stage 5	368(34.23%)
Causes of CKD	
diabetes mellitus	423(39.35%)
hypertension	256(23.81%)
Chronic glomerulonephritis	110(10.23%)
Others	286(26.61%)
Marital status	
Single	48(4.47%)
Married	858(79.81%)
Widowed	151(14.05%)
Separated	7(0.65%)
Divorced	11(1.02%)
Educational level	
Illiterate	211(19.63%)
Primary school	527(49.02%)
Junior high school	110(10.23%)
Senior high school	128(11.91%)
Junior college	41(3.81%)
University & graduate school	58(5.4%)
Current smoker	277(25.77%)

Values are expressed as mean ± SD or number (percentage).

CKD, chronic kidney disease; eGFR, estimated glomerular filtration rate; BMI, body mass index.

**Table 2:** Baseline characteristics of study population by the RDW groups

Variable	All Subjects	Low RDW (RDW< = 14.9%)	High RDW (RDW>14.9%)	p-value
	(n = 1075)	(n = 722)	(n = 353)	
sex (% male)	605(56.28%)	417(57.76%)	188(53.26%)	0.183
Age (years)	64.2±12.35	63.44±12.71	65.77±11.45	0.003
BMI (kg/ m^2^)	25.17±4.15	25.27±4.19	24.96±4.08	0.245
Current smoker	277(25.77%)	185(25.62%)	92(26.06%)	0.877
Medication prescription				
ACE inhibitor/ARB	704(65.49%)	467(64.68%)	237(67.14%)	0.426
Vitamin B12 (cyanocobalamin)	224(20.84%)	150(20.78%)	74(20.96%)	0.943
Iron preparations	169(15.72%)	82(11.36%)	87(24.65%)	<0.001
Folic acid	214(19.91%)	124(17.17%)	90(25.5%)	0.001
Calcium channel blocker	662(61.58%)	446(61.77%)	216(61.19%)	0.854
Erythropoiesis stimulating agents	209(19.44%)	114(15.79%)	95(26.91%)	<0.001
Phosphate binder	88(8.19%)	49(6.79%)	39(11.05%)	0.024
Comorbid conditions				
Cancer	102(9.49%)	60(8.31%)	42(11.9%)	0.076
Cerebrovascular disease	159(14.79%)	103(14.27%)	56(15.86%)	0.522
Chronic lung disease	158(14.7%)	99(13.71%)	59(16.71%)	0.200
Congestive heart failure	122(11.35%)	65(9%)	57(16.15%)	0.001
Coronary artery disease	269(25.02%)	166(22.99%)	103(29.18%)	0.028
Dementia	29(2.7%)	20(2.77%)	9(2.55%)	1.000
Diabetes mellitus	531(49.4%)	342(47.37%)	189(53.54%)	0.057
Hyperlipidemia	421(39.16%)	286(39.61%)	135(38.24%)	0.666
Hypertension	782(72.74%)	534(73.96%)	248(70.25%)	0.200
Liver cirrhosis	17(1.58%)	8(1.11%)	9(2.55%)	0.075
Peripheral artery disease	15(1.4%)	12(1.66%)	3(0.85%)	0.409
Laboratory data				
Creatinine (mg/dL)	3.54±2.26	3.39±2.2	3.85±2.33	0.002
eGFR (ml/min per 1.73 m^2^)	22.85±12.69	23.94±12.91	20.64±11.94	<0.001
Hemoglobin (g/dL)	10.64±2.14	11.03±2.08	9.83±2.04	<0.001
Mean corpuscular volume (fL)	90.3±7.53	91.64±5.33	87.57±10.18	<0.001
Platelet count (x10^3^/μL)	227.27±75.81	226.60±67.79	228.64±90.10	0.707
WBC count (x10^3^μL)	7.49±2.50	7.35±2.39	7.77±2.70	0.015
Albumin (g/dL)	3.7±0.65	3.8±0.61	3.47±0.68	<0.001
BUN (mg/dL)	46.1±23.14	43.28±22.02	51.85±24.3	<0.001
Cholesterol (mg/dL)	184.91±53.72	186.52±48.32	181.6±63.3	0.197
Triglyceride (mg/dL)	153.3±104.53	157.04±101.99	145.65±109.3	0.094
Ca (mg/dL)	8.78±0.67	8.83±0.63	8.68±0.75	0.001
Phosphate (mg/dl)	4.2±1.11	4.14±1.08	4.34±1.16	0.007
Calcium phosphate product (mg^2^/dL^2^)	36.62±8.96	36.24±8.59	37.42±9.63	0.051
Uric acid (mg/dL)	7.98±1.82	7.91±1.8	8.12±1.88	0.085
Proteinuria (mg/day)	1713.56±2485.78	1521.54±2130.34	2169.6±3133.9	0.008

Values are expressed as mean ± SD or number (percentage).

CKD, chronic kidney disease; eGFR, estimated glomerular filtration rate; BMI, body mass index; ACE inhibitor, angiotensin-converting enzyme inhibitor; ARB, angiotensin II receptor blocker.

### RDW and all-cause mortality

**Fig 1:**
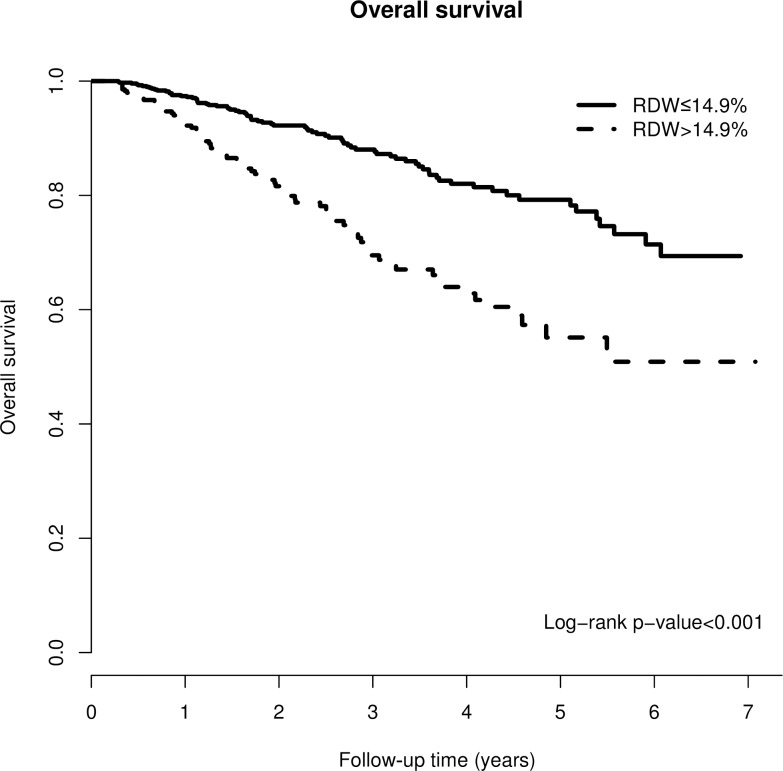
Kaplan-Meier curve of overall patient survival according to the RDW groups (log-rank test, p<0.001).

Of the 1075 participants, 158 patients (14.7%) died over a mean follow-up of approximately 2.35 years. The crude mortality rate was significantly higher in the high RDW group (high RDW group, 22.4%; low RDW group 11%, p <0.001). The Kaplan-Meier survival curve of all-cause mortality for the two RDW groups indicated that the high RDW group was associated with a higher mortality risk with a p value < 0.001 using the log-rank test (Fig 1). As shown in Table 3, the high RDW group significantly predicted the risk of all-cause mortality in both unadjusted and adjusted models. Using the adjusted model, the high RDW group was correlated with a hazard ratio of 2.19 for mortality as compared with the low RDW group (95% CI = 1.53–3.09, P<0.001).

**Table 3:** Univariate and multivariate Cox regression models of all-cause mortality, CVD mortality and infection mortality for RDW groups (RDW>14.9% versus RDW ≤ 14.9%)

	All-cause mortality		CVD mortality		infection mortality	
	Hazard ratio (95% CI)	p-value	Hazard ratio (95% CI)	p-value	Hazard ratio (95% CI)	p-value
Univariate model	2.42(1.77,3.31)	<0.001	2.45(1.26,4.76)	0.008	2.54(1.61,4.00)	<0.001
Model 1	2.29(1.67,3.13)	<0.001	2.25(1.16,4.37)	0.017	2.39(1.51,3.77)	<0.001
Model 2	2.09(1.50,2.90)	<0.001	2.25(1.16,4.37)	0.017	2.39(1.51,3.77)	<0.001
Model 3	2.18(1.56,3.03)	<0.001	2.27(1.15,4.41)	0.016	1.90(1.15,3.14)	0.012
Model 4	2.19(1.55,3.09)	<0.001	2.28(1.14,4.52)	0.019	1.90(1.15,3.14)	0.012

Univariate model: unadjusted HR for RDW.

Model 1: RDW, age, sex, BMI and smoking status.

Model 2: Model 1 plus proteinuria, albumin, BUN, calcium, cholesterol, creatinine, eGFR, hemoglobin, MCV, phosphate, platelet count, triglyceride, uric Acid, WBC count, Ca x P.

Model 3: Model 2 plus ACE inhibitor/ IARB, vitamin B12, iron preparation, folic acid supplement, calcium channel blocker, erythropoiesis stimulating agents, phosphate binder

Model 4: Model 3 plus cancer, cerebrovascular disease, chronic lung disease, congestive heart failure, coronary artery disease, dementia, DM, hypertension, hyperlipidemia, liver cirrhosis, and peripheral arterial disease

### RDW and CVD mortality

**Fig 2:**
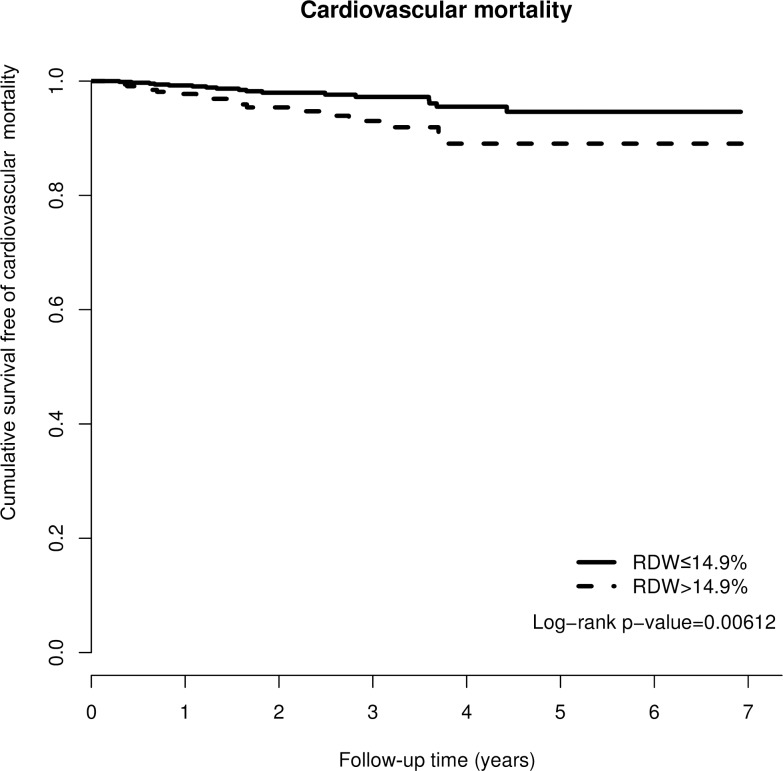
Kaplan-Meier curve of cumulative survival free of cardiovascular disease mortality according to the RDW groups (log-rank test, p = 0.00612).

In the end, 35 (3.25%) patients died of cardiovascular disease during the study period. There was a difference in the crude CVD mortality rate between the two groups (high RDW group, 5.1%; low RDW group 2.35%, p = 0.027). CVD survival difference was evident between the two groups from the Kaplan-Meier curve (Fig 2, p = 0.006). In the fully adjusted model (Table 3), the hazard ratio of CVD mortality for the high RDW group was 2.28 (95% CI = 1.14–4.25, P = 0.019).

### RDW and infection associated mortality

**Fig 3:**
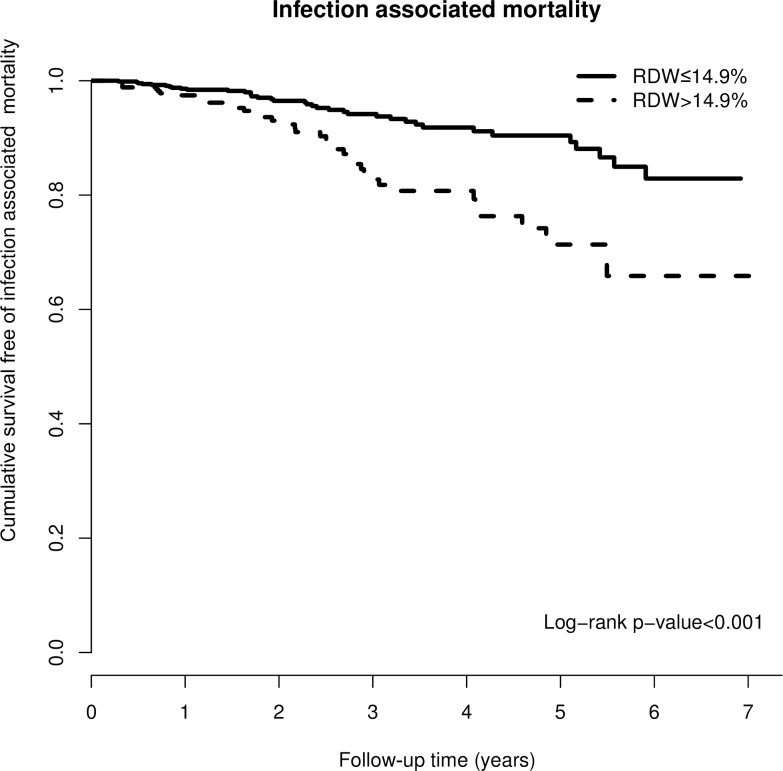
Kaplan-Meier curve of cumulative survival free of infection related mortality according to the RDW groups (log-rank test, p<0.001).

There were 75 subjects (7%) who died from infection/ sepsis in our cohort over the mean follow-up of approximately 2.35 years. There was a difference in the crude infection related mortality rate between the two groups (high RDW group, 10.8%; low RDW group 5.1%, p = 0.001). Kaplan-Meier curve showed the difference in the cumulative survival status free of infection mortality for the two groups (Fig 3, p<0.001). In the fully adjusted model (Table 3), the hazard ratio of infection mortality for the high RDW group was 1.9 (95% CI = 1.15–3.14, P = 0.012).

### The correlation of RDW with other parameters

Spearman rank correlation test was conducted to assess the correlation between RDW and other laboratory parameters (Table 4). RDW showed a positive significant association with BUN, creatinine, uric acid, WBC counts, GPT, and phosphate. A negative correlation between RDW and malnutrition makers (albumin, cholesterol) was found.

**Table 4:** Correlations of various parameters with RDW^a^

Variables vs RDW	Spearman's rho correlation	p-value
Creatinine	0.145	<0.001
eGFR	-0.143	<0.001
Hb	-0.295	<0.001
MCV	-0.203	<0.001
WBC	0.188	0.004
Platelet	0.07	0.026
Albumin	-0.246	<0.001
Bun	0.199	<0.001
Ca	-0.159	<0.001
P	0.111	<0.001
Ca x P	0.07	0.022
Cholesterol	-0.082	0.007
Triglyceride	-0.103	0.001
GPT	0.063	0.040
Uric acid	0.076	0.013

^a^Spearman’s rank test correlation

## Discussion

In the current study, we evaluated the associations between RDW and overall mortality, infection related mortality, as well as cardiovascular disease mortality in 1075 subjects with stage 3–5 CKD at a medical center in the middle Taiwan. The novel contribution of our study is that apart from other variables, RDW was found to be independently related to all-cause mortality, cardiovascular mortality and infection mortality. To the best of our knowledge, this is the first study to unveil the association of RDW with mortality in CKD patients.

The use of RDW has been limited as an auxiliary parameter in the diagnosis of anemia until recently when it was demonstrated to be an important predictor of prognosis in both acute and chronic clinical settings. For example, RDW was one of the independent predictors for 30-day mortality and length of hospital stay in patients hospitalized for community-acquired pneumonia (8). The study concluded that the use of RDW improved the prognostic performance of the severity scale for pneumonia. Another study reported that RDW can predict post-discharge patient survival following critical illness [12]. For patients with chronic illness, the correlation of RDW with mortality has been repeatedly studied in patients with heart disease. For example, a large cohort study of chronic heart failure population showed that RDW predicted mortality [2]. RDW was also linked with the severity and progression and first hospitalization in patients with heart failure [13]. Furthermore, several epidemiologic studies have also observed the association of RDW with mortality in large unselected community-based populations [14, 15]. However, the exact mechanisms linking RDW to negative outcomes have not yet been fully elucidated. It is commonly accepted that anemia is one of the key factors leading to greater variation in erythrocyte size. Mechanisms causing anemia could affect RDW at an earlier stage before the hemoglobin level falls into the anemia range [16]. Previous evidence also suggested that iron deficiency may first raise RDW, followed by abnormalities in other blood cell counts before the anemia occurs [17]. Thus, elevated RDW may represent the complex interaction in the process of anemia before clinically apparent anemia occurs.

Anemia and hemoglobin levels have been demonstrated to independently predict mortality in different patient populations [18, 19]. Anemia is well known to be prevalent in CKD patients. The causes of renal anemia include decreased erythropoietin production, increased inflammation, mineral bone disorder, and shortened erythrocyte life span, as well as iron deficiency of either relative or absolute form. The presence of anemia also contributed significantly to mortality in CKD patients [20]. In our study, adjustment for hemoglobin did not abrogate the significant effect of RDW, which means that the association is independent of anemia in the CKD population. Furthermore, the relationship between RDW and unfavorable outcomes may be partly explained by the theory speculated by Bion who considered RDW to be the patient’s physiologic reserve [21]: when physiologic reserve is exhausted or impaired, immature erythrocytes of heterogeneous size emerge in the peripheral circulation and result in higher RDW. Therefore, it is possible that even a small change in RDW may reflect the subtle disorder of patients’ health which can subsequently lead to adverse outcomes.

Some potential pathophysiologic pathways have been suggested to explain why high RDW increase the risk of adverse outcomes, with inflammation and malnutrition being the two most common mechanisms among them. In a study of 786 community-dwelling elderly women, Semba et al. reported that two antioxidant factors, total carotenoids and selenium, were correlated with RDW [22]. However, since the significance was attenuated after adjusting for interleukin-6, the relationship between RDW and oxidative stress was mediated in part by inflammation. A recent study of 195 patients with heart failure demonstrated a significant association between RDW and inflammation and nutritional status and it was found that RDW independently predicted mortality [23]. RDW has also been correlated with soluble tumor necrosis factor receptor and CRP in the settings of rheumatoid arthritis and heart failure [24, 25]. A recent study of coronary artery disease patients has also showed an association of RDW with CRP, in which higher RDW was related to worse survival [26]. A condition of chronic inflammation might be related to ineffective erythropoiesis through iron metabolism or an inhibitory effect of pro-inflammatory cytokines on bone marrow, or to the increased erythrocyte destruction [27]. As for CKD patients, Solak et al. reported that there was a strong association of RDW with serum CRP and albumin independent of other significant covariates [11]. Feng et al. found that RDW was positively correlated with inflammation and malnutrition in a cohort of 1293 peritoneal dialysis patients, and it emerged as an independent predictor of cardiovascular mortality [28]. A similar association was also observed in hemodialysis patients [29]. Moreover, RDW was reported to be related to inflammation, nutrition and mortality in kidney transplant recipients [4]. The above evidence indicates the robust association of RDW with inflammation and under-nutrition might lead to worse clinical outcomes in patients with elevated RDW levels.

Beyond inflammation and malnutrition, there is little data in the literature investigating the underlying causes for RDW with mortality. Some other possible mechanistic explanations were proposed for the association between RDW and adverse outcomes. Kalay et al. found RDW independently predicted slow coronary flow, a condition characterized by the delayed passage of contrast without structural coronary disease [30]. A recent investigation of 367 patients with CKD stage 1 to 5 unveiled an independent association of RDW with endothelial dysfunction assessed by flow-mediated dilatation and carotid intima media thickness [11]. In other words, RDW was closely linked with the earliest and advanced stages of atherosclerosis, which accounts for the disproportionately high cardiovascular burden in CKD patients. Celik et al. showed an independent association of RDW with left ventricular filling pressure among patients with diastolic heart failure [31]. Furthermore, Leszek et al. unveiled the close relationship between RDW and left ventricular diastolic dysfunction in CKD patients of advanced stage [32]. In addition, elevated RDW was related to reduced erythrocyte deformability, which can lead to impaired microcirculation [33]. The resultant hypoxia may cause increased mortality in patients with high RDW.

The strengths of our study included its large sample size and comprehensive data set. However, as any retrospective observational study, our study had several limitations that needed to be mentioned. First, a cause-and-effect relationship cannot be clearly inferred by the retrospective design. Moreover, the underlying mechanisms also cannot be answered. Second, the single-center design also limited the generalization of the results of the present study to the whole CKD population. Third, despite the adjustment for many potential confounders, the possibility of residual important confounders that were not evaluated in our study like endothelial dysfunction remains to be present.

In conclusion, this is the first study to date indicating a negative effect of RDW on patient survival in stage 3–5 CKD patients. Since RDW was associated with mortality independent of other clinical covariates, it should be included among those nontraditional risk factors for mortality. RDW is an available and free-of-cost parameter because it is routinely reported in standard complete blood cell counts. Our results propose it may be used as a useful prognostic indicator in the clinical risk assessment in CKD patients. More medical attention and care should be paid to those patients with high RDW. Further investigations are needed to elucidate the pathophysiological mechanisms responsible for the relation of RDW with mortality.
